# Degree of locking to network activity of neurons with similar movement tuning in the motor cortex of awake, behaving rats differs by layer

**DOI:** 10.1186/1471-2202-12-S1-P165

**Published:** 2011-07-18

**Authors:** Kensuke Arai, Emi Takakuda, Takeshi Takekawa, Yoshikazu Isomura, Tomoki Fukai

**Affiliations:** 1 RIKEN Brain Science Institute, Wako, Saitama, Japan; 2 Department of Complexity Science and Engineering, University of Tokyo, Kashiwa, Chiba, Japan; 3 Brain Science Institute, Tamagawa University, Machida, Tokyo, Japan

## 

The rat motor cortex, like other areas of the mammalian neocortex, is organized in a 6-layered structure. The synaptic organization has been studied in the past by various *in vitro* techniques [1,2], and the movement-specific tuning properties of the neurons in forelimb area of awake, freely-behaving rats has also recently been studied [3]. In this last study, rats were trained to push down on a lever for > 1 second, and to pull back up to receive a water reward. The tuning properties of the neurons were studied by triggering on the pull-up of the lever, and neurons were classified according to the modulation of the firing rate relative to the pull-up trigger as pre-movement, movement, post-movement, hold-related, movement off, and non-related. It has been revealed that there is little difference between the tuning properties of cells in the various layers; all layers contain pretty much all tuning types, although the average firing rate of cells is higher in the deeper layers. Considering only the rate information, there does not seem to be much difference in the computations being carried out in the various layers.

The local field potential (LFP) is thought to represent summed local synaptic currents, and as such, can be considered as containing information about the activity of the neural circuits, both cortical and extra-cortical. In this work, we pose the question whether there is really no laminar organization in the computations being carried out in the various layers of the forelimb area of M1. We analyzed the data presented in [3], obtained using the juxtacellular method of recording spikes from single, identifiable units in addition to multi-channel laminar LFP recordings from layers 1~6. Using this data, we analyzed the spike-LFP relationship using sliding, variable-width windows in which the LFP spike-triggered average (STA) was calculated for times between -1000 ms and 300 ms preceding and following the onset of a lever pull. The STAs were then assigned a z-score by comparing against an ensemble of STAs calculated using shuffled spike times (sSTA), effectively giving a number representing the significance of precise spike timing information. This was carried out for each of the 9 LFP channels, giving us a spatio-temporal plot of a given neuron’s relationship to the LFP. The resulting plots show a clear difference between L2/3 and L5/6 neurons. L2/3 neurons show fairly uniform and strong spike-LFP relationship throughout the analysis period, suggesting that these neurons are strongly locked to the underlying LFP regardless of the phase of movement. In contrast, cells in L5/6 often showed weaker spike-LFP relationship, and more modulation of strength throughout the analysis period, suggesting a more variable degree of spike precision to some underlying network activity.
